# Physiology

**Published:** 1824-03-01

**Authors:** 


### Physiology.

1. *Physiology of the Urinary Secretion.* It is acknowledged that the physiology of secretion is involved in deep obscurity. Some think that the glandular organs merely strain or separate the fluids from the blood—others believe that entirely new products are formed in the secerning vessels, and that no part of the peculiar secretion previously existed in the blood. A third party conceive that the *elements* of the various secretions pre-existed in the general circulation, and that they are combined, each according to its kind, in the glandular apparatus appointed for that purpose. This appears to be the more probable doctrine.

In respect to the urinary secretion, it is well known that, in general, there are between twenty and thirty different ingredients in it; but its characteristic component part is termed urea, a gellatinous animal substance always present in healthy urine.

It was to ascertain the state and comparative proportion of this in the blood, that Messrs. Prevost and Dumas have made many experiments—or rather repeated those previously made by M. Richerand —namely, the extirpation of the kidneys, from dogs and cats, rabbits not being found to bear these experiments well. One kidney is to be removed first, and when the wound is healed, (about 15 days,)

the other organ is to be taken away. The health is nowise affected by the first operation. In about three days after the removal of the second kidney, copious liquid brown stools, and vomiting of the same materials occur, with great disturbance of the system, as rapid and small pulse, short and laborious respiration. The animal dies between the fifth and ninth day. On dissection, effusions of clear limpid serum are found in the ventricles of the brain—the bronchiæ filled with mucus—the liver inflamed—the intestines containing liquid fæces—the urinary bladder strongly contracted.

It was estimated by our authors that a dog in health formed a drachm of urea daily, and it was found that five ounces of blood from a dog which had been deprived of its kidneys yielded more than a scruple of urea. The experiments of these gentlemen lead them to believe that the urea is *eliminated merely* by the kidneys, in proportion as it is formed—and consequently that it accumulates in the circulation when the kidneys are removed. In this case, therefore, we must suppose that the urea is formed independent of the action of the kidney. *Where* it is formed, is not yet ascertained. It is known that in certain diseases of the liver, the urea disappears from the urine. If we apply this doctrine to diabetes, we may suppose that the saccharine matter is formed elsewhere than in the kidneys, and merely thrown off by them in solution.

2. *The Circulation.* Is it certain that the ancients considered the arteries to be the conductors *merely* of the animal spirits? How does the following passage, Pauli Æginetæ, quadrate with this opinion?

"Hæ arteriæ vero oblonga sunt vasa velut venæ, et duas tunicas habent, tum propter relatum motum, tum quod *sanguinem et spiritum* continent: et enascuntur ex corde, et disperguntur per omnes corporis partes."—*De Pulsibus, cap.* xii.

3. *Nervous System.** We are glad to see the commencement of a series of experimental researches on the functions of the nervous system, under the able auspices of Dr. Breschet and his associates. This first memoir is on the subject of digestion, and it is gratifying to find that the experiments of our Parisian neighbours have confirmed those of our own distinguished countryman, Dr. W. Philip, in respect to the division of the eighth pair of nerves.

The experiment is one of the oldest on the records of physiology. In all ages it was observed that the division of the par vagum disturbed the functions of the lungs and stomach, but it is to this last phenomenon that the attention of our authors is directed. Most experimenters have remarked that those animals who suffered the section of the eighth pair were harrassed with nausea and vomiting—a

* Recherches Experimentales sur les Fonctions du Systeme Nerveux. 1[er] Memoire; de l'Influence du Systeme Nerveux sur la Digestion Stomachale. Par M. M. Breschet, Edwards, et Vavasseur. Archives Generales.

circumstance that proved the influence of these nerves on the stomach. On this point all are agreed—but not so on the precise effects of this section on the actual process of digestion. Ducrotay de Blainville concluded from experiments, that "by the division of these nerves the digestive powers of the stomach were totally annihilated." Mr. Brodie came to the same conclusion, though with a different object in view. He found the aliment undigested seven hours after the nerves had been cut. Legallois and Dupuy arrived at the same results. But it was reserved for our countryman, Dr. W. Philip, to push these experiments much farther—by proving, as our readers know, that when the nerves were divided, digestion might still be carried on, provided a stream of electricity were conveyed along the lower portion of nerve to the stomach. Our readers are also well aware that, in consequence of simply dividing the pneumo-gastric nerves, without removing a piece, or keeping the divided extremities well apart, the experiments were vitiated, for the digestion, under such circumstances, went on, though in a somewhat imperfect manner. All these experiments have now been repeated by the authors in question, in varied modes, and on animals of different species, and the whole results are confirmatory of the statements of Dr. W. Philip. Instead of rabbits, however, our experimenters had recourse to much larger animals, as the horse, the dog, &c. We shall relate the following experiment on the first named animal.

Three horses were selected, and all fed on oats after long abstinence. In one, the pneumo-gastric nerves were divided and a portion cut away—one horse was left untouched, and the third was managed in the following manner. The right par vagum was laid bare and between two and three inches of it excised. The left was also divided and a portion of its lower extremity isolated, and encircled with a bit of lead, to which was fastened a wire that led to a galvanic apparatus. The electric circle was completed by introducing into the abdomen, below the stomach, the point of a conductor communicating with the opposite pile. Slight but constant contractions in the panniculus carnosus indicated the uninterrupted stream of electricity for seven hours. Early in the operation it was necessary to make a large opening in the horses' tracheæ that were operated on, to prevent suffocation.

The results of these experiments were very unequivocal. The digestion was complete in the animal untouched. In the horse subjected to galvanism the stomach was but little distended, and contained but a small quantity of fluid and of corn a little acted on. All the rest had passed into the cæcum, where it was found, for the most part, reduced to chyme. In short, the appearances were but little different from those in the sound animal. On the other hand, the stomach of the horse, whose pneumo-gastric nerves had been excised, was greatly distended, containing much clear and yellowish liquid, and triple the quantity of corn remaining in the stomach of the galvanized animal. The greater part of the oats had suffered little or no alteration—a small quantity only appearing about half digested. In the cæcum was found only an aqueous fluid, with some debris of straw, evidently the remains of a former digestion. Thus it was clear that in this horse no part of the corn eaten after a long fast had passed the pylorus, while the whole of that eaten by the sound horse, and two-thirds of that eaten by the galvanized animal, had advanced into the intestines. These experiments were repeated in the same manner on dogs and other animals with similar results.

From the above and many other experiments, which we need not detail here, our authors have come to the following conclusions.

1st. The simple section of the pneumo-gastric nerve, without loss of substance or disturbance of relative position does not materially interrupt the process of digestion, but only renders it more slow.

2d. The section of the nerves, with loss of substance, diminishes considerably (and far more than the simple section) the digestive action of the stomach; but it does not entirely abolish that process.

3d. The section or destruction of a part of the spinal marrow, or the removal of a portion of the cerebrum, acts in like manner to the above on the digestive process.

4th. Narcotics administered so as to produce coma, equally diminish the power of digestion.

5th. Every thing that diminishes the sum total of nervous influence going to the stomach, enfeebles proportionally the process of digestion in that organ.

6th. Finally, when the digestion is almost completely suspended by excision of a portion of the pneumo-gastric nerves, we can, by means of galvanism, re-establish the digestive process in the stomach, and convert into chyme the aliment introduced into that organ, almost as quickly and completely as under ordinary circumstances of health.

These conclusions differ little or nothing from those at which Dr. Philip had arrived; but still the experimenters in question do not come completely into the opinion of our countryman, that the process of digestion is entirely owing to nervous influence. They acknowledge, however, that it is the most important of all the agents in this operation.

